# Real‐Time 3‐Dimensional Dynamics of Functional Mitral Regurgitation: A Prospective Quantitative and Mechanistic Study

**DOI:** 10.1161/JAHA.113.000039

**Published:** 2013-06-21

**Authors:** Yan Topilsky, Ori Vaturi, Nozomi Watanabe, Valentina Bichara, Vuyisile T. Nkomo, Hector Michelena, Thierry Le Tourneau, Sunil V. Mankad, Soon Park, Mary Ann Capps, Rakesh Suri, Sorin V. Pislaru, Joseph Maalouf, Kiyoshi Yoshida, Maurice Enriquez‐Sarano

**Affiliations:** 1 Division of Cardiovascular Diseases, Tel Aviv Medical Center, Rochester, MN (Y.T.); 2 Division of Cardiovascular Diseases and Internal Medicine, Mayo Clinic, Rochester, MN (O.V., V.B., V.T.N., H.M., T.T., S.V.M., M.A.C., S.V.P., J.M., M.E.S.); 3 Department of Cardiology, Kawasaki Medical School, Japan (N.W., K.Y.); 4 Division of Cardiothoracic Surgery, Mayo Clinic, Rochester, MN (S.P., R.S.)

**Keywords:** echocardiography, mitral regurgitation, mitral valve annulus

## Abstract

**Background:**

Three‐dimensional transthoracic echocardiography (3D‐TTE) with dedicated software permits quantification of mitral annulus dynamics and papillary muscle motion throughout the cardiac cycle.

**Methods and Results:**

Mitral apparatus 3D‐TTE was acquired in controls (n=42), patients with left ventricle dysfunction and functional mitral regurgitation (LVD‐FMR; n=43) or without FMR (LVD‐noMR, n=35). Annulus in both normal and LVD‐noMR subjects displayed saddle shape accentuation in early‐systole (ratio of height to intercommissural diameter, 10.6±3.7 to 13.5±4.0 in normal and 9.1±4.3 to 12.6±3.6 in LVD‐noMR;* P*<0.001 for diastole to early‐systole motion, *P*=NS between those groups). In contrast, saddle shape was unchanged from diastole in FMR patients (10.0±6.4 to 8.0±5.2; *P*=NS,* P*<0.05 compared to both other groups). Papillary tips moved symmetrically towards to the midanterior annulus in control and LVD‐noMR subjects, maintaining constant ratio of the distances between both tips to midannulus (PtAR) throughout systole. In LVD‐FMR patients midsystolic posterior papillary tip to anterior annulus distance was increased, resulting in higher PtAR (*P*=0.05 compared to both other groups). Mechanisms of early‐ and midsystolic FMR differed between different etiologies of LV dysfunction. In patients with anterior MI and global dysfunction annular function and dilatation were the dominant parameters, while papillary muscle motion was the predominant determinant of FMR in patients with inferior MI.

**Conclusions:**

Inadequate early‐systolic annular contraction and saddle‐shape accentuation in patients with impaired LV contribute to early–mitral incompetency. Asymmetric papillary tip movement towards the midanterior annulus is a major determinant of mid‐ and late‐systolic functional mitral regurgitation.

## Introduction

Functional mitral regurgitation (FMR) occurs despite structurally normal mitral leaflets as a consequence of left ventricular (LV) dysfunction.^1^ FMR is poorly characterized because it is often silent,^2^ but echocardiography has demonstrated its high frequency.^3^ Severe FMR portends poor hemodynamics^4^ and prognosis^5–6^ underscoring the importance of comprehending its determinants. FMR is undeniably associated with LV remodeling and enlargement, but has been variably attributed to global LV dilatation,^7^ mitral annulus enlargement, or local LV remodeling (apical and posterior displacement of papillary muscles) leading to excess valvular tenting.^1,8^

There are some suggestions that FMR is dynamic under different conditions^9–10^ and during the cardiac cycle,^9,11–12^ but the quantitative dynamic changes of FMR and mitral apparatus during the cardiac cycle remain poorly defined. Recent advances in noninvasive Doppler echocardiography allow reliable instantaneous assessment of regurgitant flow throughout the cardiac cycle^7,9,13–14^ and with the advent of real‐time 3‐dimensional (3D), full‐volume acquisition, new insights into physiology of mitral apparatus, particularly mitral annulus, have been obtained.^15^ However, for FMR assessment, transesophageal 3D echocardiography is limited because FMR may diminish or even disappear during the procedure^9^ and because patients with LV dysfunction, with or without FMR, are rarely clinically referred to the test, so that the limited sample examined may be skewed. With the introduction of novel real‐time 3D, full‐volume acquisition with transthoracic echocardiography (RT3DE), high‐quality, high‐frame‐rate, imaging of the entire annular and left ventricular volume over full cardiac cycles is now possible.^15–17^ This advance, coupled with quantitative software measurements and quantitative analysis of mitral regurgitation throughout the cardiac cycle heralds a new era of physiological assessment of mitral valvular, subvalvular, and annular function^18–19^ under physiologic conditions. Such analysis is essential because FMR independently predicts poor outcome and thus is a major target of medical, surgical, or newly introduced percutaneous techniques.

Thus, we aimed at investigating mitral annular size, shape, and motion, and the dynamic relationship between mitral annulus and papillary muscles throughout the cardiac cycle using RT3DE in patients with FMR versus control subjects and patients with low ejection fraction but no FMR.

## Methods

### Patient Population

All patients enrolled underwent a clinically indicated echocardiogram during which they were offered participation, provided informed consent, and underwent RT3DE. We primarily enrolled 150 patients in the study, but excluded 30 (20%) patients based on poor image quality leaving 120 patients included in our cohort. There were no systematic differences between patients excluded or included in our analysis and patients excluded had similar age, gender, and ejection fraction to those included in the analysis (*P*>0.2 for all comparisons). The excluded patients were represented in all groups: 6 in the normal group (6/48; 12.5%), 12 in the LVD‐noFMR group (12/56; 21%) and 12 in the LVD‐FMR group (12/46; 26%), *P*=0.2. All patients had a structurally normal mitral valve by echocardiographic evaluation diagnosed from January to June 2010 and were enrolled prospectively. Patients enrolled as controls had a completely normal echocardiogram and no or trivial MR. Patients with left ventricular remodeling (LVD), defined either as reduced overall LV function with ejection fraction (EF) <50% or as extensive regional wall motion abnormalities despite EF>50%, were also enrolled as cases of interest. Cases were classified as LVD with FMR if they had at least holosystolic mild FMR, quantifiable by the Doppler‐echocardiographic methods (LVD‐FMR). The patients with LVD with no mitral regurgitation or with only trivial early‐or late–partial‐systolic regurgitation (LVD‐noMR) were also enrolled and underwent similar Doppler‐echocardiographic and 3D protocol. We have defined functional MR based on quantified MR with an effective regurgitant orifice of at least 0.1 cm^2^. Trivial MR was defined whenever regurgitation was barely detected rendering it nonquantifiable. Exclusion criteria were ongoing or recent (within 3 months) acute coronary syndrome, bacterial endocarditis, presence of more than mild mitral stenosis or aortic valve disease, moderate or more tricuspid regurgitation, pericardial or congenital heart disease. The cause of LVD was defined as ischemic based on a history of myocardial infarction, a scar or myocardial infarction by echocardiography, or coronary angiography as ascertained by the consultant cardiologist. Clinical characteristics were also recorded as defined by the consultant cardiologist. The Institutional Review Board of our institution approved the study and in view of the low study risk required only oral informed consent.

### Trans‐Thoracic 2D and 3D Echocardiography

All of the echocardiographic examinations were performed by using the same ultrasound equipment (iE33, Philips Medical Systems) with the S3 probe for 2‐dimensional (2D) images and X4 or X3‐1 probe for real‐time 3D images. All patients underwent a standard 2D echocardiographic examination with determination of left ventricular ejection fraction, end‐systolic and end‐diastolic dimensions, and left atrial volume according to American Society of Echocardiography recommendations.^20^ For patients with FMR, quantification of overall FMR used either the proximal isovelocity surface area (PISA) method or quantitative Doppler or both following the American Society of Echocardiography recommendations to calculate regurgitant volume (RVol) and effective regurgitant orifice (ERO). The proximal isovelocity surface area method was also used in all patients with FMR to perform instantaneous ERO assessment sequentially in early‐, mid‐, and late‐systole using the corresponding MR velocities and flow convergences. Early‐systole was identified as immediately following onset of mitral closure, late‐systole at or immediately before aortic closure, and midsystole midway between these frames. We subsequently calculated RVol by using the TVI in the first‐, mid‐, and last‐third of systole. For example, to calculate the ERO in early‐systole (ERO_early_) we used the formula:
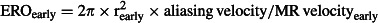
1where r_early_ and MR velocity_early_ are the radius of the hemispherical shell and mitral regurgitant velocity at early‐systole, respectively. We traced the integral of mitral regurgitant jet of the first‐third of systole (MR TVI_early_) and calculated early–systolic‐RVol as RVol_early_=ERO_early_×MR−TVI_early_. We used the same formulas to calculate ERO and RVol at mid‐ and late‐systole. In patients with no or trivial FMR by color Doppler, RVol and ERO was assumed as null.

Transthoracic 3D echocardiographic volumetric images (full‐volume mode) were obtained from the apical view after 2D verification of appropriate positioning and quality of image. The volumetric frame rate was 15–20 frames/sec with imaging depth usually around 15 cm. RT3DE was similarly performed in controls. LVD‐FMR, and LVD‐noMR patients. Full‐volume 3D data sets were digitally stored, transformed into Cartesian coordinates, and transferred to a workstation with a custom software system (Real View^®^, YD, Ltd).

### Quantification of Mitral Valve Coaptation by 3D Echocardiography

**Figure 1.**
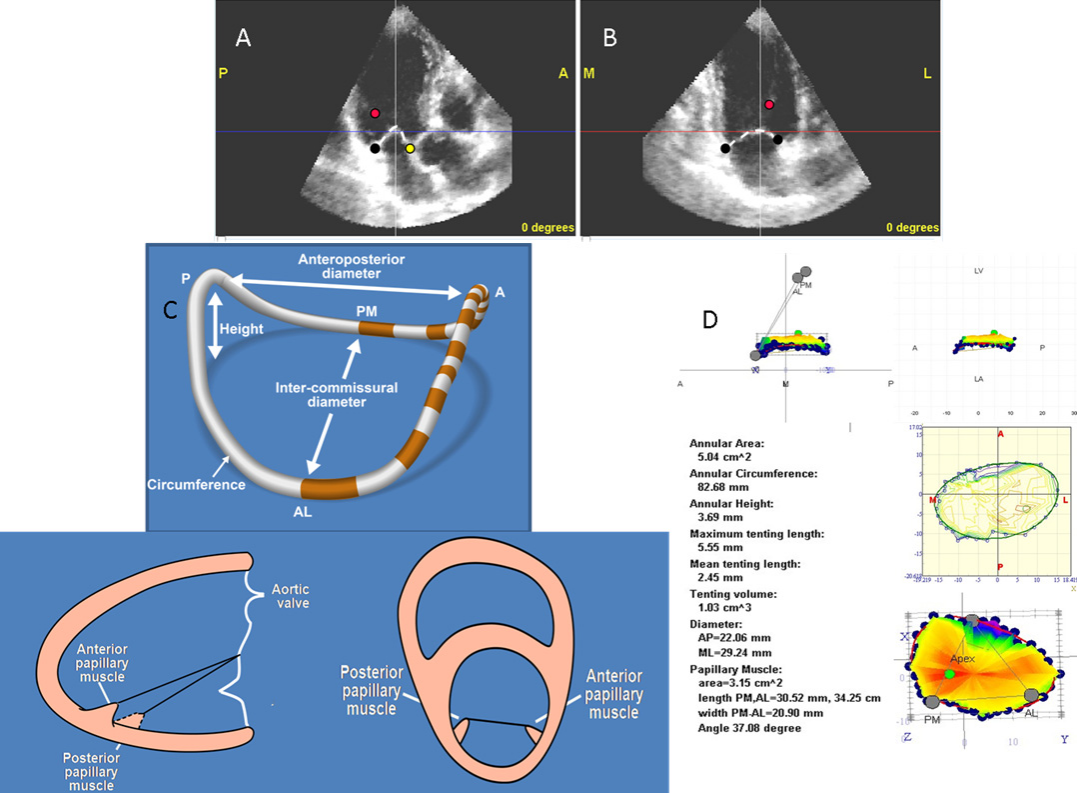
Three‐dimensional (3D) data are automatically cropped into 18 radial planes. A and B, Annular (black circles) and leaflets tracing (broken white line) are semiautomatically defined and manually adjusted in each radial plane. Posterior and anterior papillary tips are marked manually (red circles) when clearly visible by examination of all radial planes. Midanterior mitral annulus (yellow circle) was defined in cross‐sectional views after complete annulus marking to ensure appropriate positioning. C, Schematic representation of a 3D reconstructed mitral annulus and the relationship between papillary muscles and between the papillary muscles and the annulus. D, Standard measurements are illustrated in the report produced by the dedicated quantification software. All spatial positioning and measurements were performed 6 times during the cardiac cycle, in early‐, mid‐, and late‐diastole and early‐, mid‐, and late‐systole. PM indicates papillary muscle; AP, anterior papillary; AL, antero‐lateral; PM, postero‐medial; ML, medial‐lateral (intercommisural diameter); AP, antero‐posterior.

The quantification software was used to analyze volumetric images as previously described.^21–22^ Once the entire 3D dataset was captured, steps of quantitation involved first defining, in cross‐sectional planes the LV long‐axis through the mitral annulus center and mitral annular anterior‐posterior and intercommissural axes. Then, 3D data were automatically cropped into 18 radial planes spaced 10 degrees apart (Figure 1). Annular marks and leaflets tracings were semi‐automatically defined and manually adjusted in each radial plane. Then, posterior and anterior papillary tips were marked manually where clearly visible by examination of all radial planes. Midanterior mitral annulus (valvular fibrosa) was defined in cross‐sectional views after complete annulus marking to ensure appropriate positioning. All spatial positioning and measurements were performed 6 times during the cardiac cycle, in early‐, mid‐ and late‐diastole and early‐, mid‐, and late‐systole. Early‐diastole was identified just after mitral valve opening, late‐diastole as preceding mitral closure and middiastole as midway between these frames. Early‐systole was identified as immediately following onset of mitral closure, late‐systole at or immediately before aortic closure, and midsystole midway between these frames. From these positioned structures, anatomic 3D images of mitral apparatus were reconstructed for display 6 times in each cardiac cycle with, at each phase measurement of mitral annular area, annular circumference, antero‐posterior diameter, intercommissural diameter, leaflet maximal tenting volume, papillary muscle area (the area of the triangle formed between both papillary tips and midanterior annulus—PtAT), length of posterior papillary tip to midanterior annulus (PPM‐AA length), length anterior papillary tip to midanterior annulus (APM‐AA length), interpapillary tips distance (PAP‐width), and acute angle of the triangle formed between papillary tips and midanterior annulus (inter‐PAP angle). Annular height was measured as well and was defined as the instantaneous maximal vertical distance between the highest (anterior or posterior) and lowest (antero‐lateral or postero‐medial) points and was used to compute instantaneous ratio of annular height to intercommissural diameter, a measure of annular saddle shape.^17^ Deeper mitral annular saddle shape is characterized by more apical position of medial and lateral aspects of the annulus, whereas the anterior and posterior aspects remain basal in position (higher ratio of annular height to intercommissural diameter). The ratio of PPM‐AA length to APM‐AA length was calculated as a measure of symmetric position of papillary muscles in regard to the mitral annulus center. Variability of measurement was determined by repeating measurements on stored 3D data sets at least 1 week after initial measurement by the same observer (intraobserver) and a different observer (interobserver).

### Statistical Analysis

Group statistics were represented by mean mean±SD for continuous variables and percentages for categorical variables. The main analyses focused on comparing apparatus characteristics between the groups LVD‐FMR, LVD‐noMR, and controls. The dynamic changes of each measurement of the mitral apparatus or papillary muscle to mitral annulus relationship were analyzed using the repeated‐measures model for the entire cycle and paired *t* test. Group comparisons of baseline characteristics, of time‐defined mitral apparatus measurements (such as overall, diastolic, and systolic average dimensions), and of percent changes in tenting volume and tenting height used ANOVA with post‐hoc Tukey test for comparisons between groups. Multivariable analyses used stepwise regression models with early‐ or midsystolic ERO as dependent variables, early‐ or midsystolic annular area, tenting volume, ratio of annular height to intercommissural diameter, and ratio of PPM‐AA length to APM‐AA length, including interaction terms for the different etiologies for systolic dysfunction (inferior MI, anterior MI or dilated cardiomyopathy). Model fit validations were performed by graphical residual analysis. Variability of measurements was assessed in 15 randomly selected patients (5 in each group) by the Bland‐Altman method and by the within‐subject coefficient of variation. Using the Bland‐Altman method, we calculated 95% confidence intervals of the variability range for the different parameters. The study was powered to detect at least a 3.5% difference in saddle shape early‐systolic accentuation between the LVD‐FMR group and other groups, with at least 80% power to detect the difference, and a *P‐*value <0.05.

## Results

### Study Population

Characteristics of the 43 LVD‐FMR patients are compared with those of the 35 LVD‐noMR and 42 controls in Table 1. As expected, LVD‐FMR patients were older (67.9±15.9 versus 62.4±16.8 versus 57.3±15.6 years; *P*=0.01), were more often men (76.7% versus 76.7% versus 47.6%; *P*=0.009), and had worse symptoms (NYHA III/IV 25.6% versus 2.8% versus 2.4%; *P*<0.0001). The LVD cause was more often associated with ischemic heart disease in LVD‐FMR than LVD‐noMR (69.7% versus 34.2%; *P*<0.0001), and was associated with higher wall motion scores. Forty‐four (37%) patients in our cohort had intraventricular dys‐synchrony. Thirteen (11%) patients had left bundle branch block, 7 (6%) had right bundle branch block, 18 (15%) had permanent pacemaker with right ventricular pacing, and all other 6 patients had QRS >120 ms. The prevalence of intraventricular dys‐synchrony was different between the groups (3 [7%] patients in the normal group, 17 [50%] in the LVD‐noMR group, and 24 [54%] in the LVD‐FMR group; *P*<0.0001). As expected, larger left atrial dimensions, lower cardiac index, and evidence of higher left and right filling pressure (higher E‐wave velocity, shorter deceleration time, higher E/e' ratio, lower pulmonary vein S‐wave velocity and systolic pulmonary pressure) were observed in LVD‐FMR compared with LVD‐noMR, and control subjects. Conversely, ventricular dimensions in LVD‐FMR patients compared with LVD‐noMR patients were similar (and worse than in controls). MR presence and degree by qualitative assessment was trivial in 11 control subjects and absent in 31, trivial in 14 LVD‐noMR patients and absent in 21, while all patients with LVD‐FMR were considered to have mild‐moderate to severe MR. In patients with LVD‐FMR the mean RVol was 36±13 mL/beat and mean ERO 0.23±0.08 cm^2^. All patients had structurally normal mitral leaflets and LVD‐FMR valvular alterations were considered of Carpentier type IIIb (systolic restriction).

**Table 1.** Participant Characteristics

Variable	Controls (n=42)	LVD‐NoMR (n=35)	Functional MR (n=43)	*P* Value
Clinical				
Height, m	1.7±0.09	1.7±0.09	1.7±0.1	0.09
Weight, kg	76.1±19.2	86.4±14.8*	80.5±16.8	0.03
Body mass index	26.0±4.9	27.9±4.0	26.7±4.1	0.14
Hypertension, n (%)	17 (40)	12 (35)	22 (51)	0.3
Diabetes mellitus, n (%)	4 (10)	4 (11)	16 (37)†	0.002
Hyperlipidemia, n (%)	11 (26)	17 (48)	15 (35)	0.12
Systolic murmur, n (%)	11 (26)	7 (20)	29 (67)║¶	<0.0001
Echocardiography				
Systolic blood pressure, mm Hg	125.2±21.7	120.0±20.6	113.1±16.5‡	0.02
Diastolic blood pressure, mm Hg	71.2±12.0	69.1±9.8	67.1±12.8	0.27
Left ventricle				
Ejection fraction, %	64.2±4.7	35.7±11.3§	30.3±11.1 † ¶	<0.0001
WMS	1[1, 1]	2[1.5, 2]§	2.3[2, 2.5]║¶	<0.0001
Normal/Inferior/Anterior/Global, %	NOR:100; INF:0; ANT:0; DCM:0	NOR:0; INF:16; ANT:18; DCM:66	NOR:0; INF:25; ANT:44; DCM:31	<0.0001
Diastolic dimension, mm	47.8±4.4	59.4±6.8§	62.0±8.7¶	<0.0001
Systolic dimension, mm	30.1±4.0	47.0±9.0§	51.1±8.6¶	<0.0001
LAVI, mL/m^2^	30.2±8.6	39.1±12.0*	56.2±15.2║¶	<0.0001
Cardiac index	3.3±0.6	2.9±0.5*	2.6±0.8¶	<0.0001
Mitral inflow E wave	0.8±0.22	0.7±0.25	1.0±0.3║¶	<0.0001
Mitral inflow DT, ms	216.1±48.4	206.5±56.7	152.1±34.9║¶	<0.0001
E/e' ratio	10.5±3.0	14.1±7.1	25.7±13.3║¶	<0.0001
Pulmonary vein S wave, m/s	0.6±0.16	0.6±0.12	0.39±0.16 † ¶	<0.0001
Right ventricle systolic pressure, mm Hg	28.6±6.0	32.3±9.4	51.5±14.0║¶	<0.0001

LVD‐noMR indicates left ventricle dysfunction without functional mitral regurgitation; MR, mitral regurgitation; WMS, Wall Motion Score; NOR, normal function; INF, inferior MI; ANT, anterior MI; MI, myocardial infarction; DCM, dilated cardiomyopathy; LAVI, left atrial volume index; DT, deceleration time; EF, ejection fraction.

* *P*<0.05,

^§^ *P*<0.001 low EF vs normal.

^†^ *P*<0.05,

^║^ *P*<0.001 functional MR vs low EF.

^‡^ *P*<0.05,

^¶^ *P*<0.001 functional MR vs normal.

### Mitral Valvular and Regurgitation Dynamics

The time course in systole of dynamic changes in valvular tenting height and volume in all 3 groups of patients and MR in LVD‐FMR patients is shown in Table 2. In normal controls, the mitral valve position is dynamic with mitral valve tenting height and volume decreasing after early‐systole (*P*<0.0001) but with no significant change between mid‐ and late‐systole (all *P*>0.08). Similar patterns were observed in LVD‐noMR (*P*<0.0001) with no further change between mid‐ and late‐systole (all *P*>0.26) and in LVD‐FMR (*P*<0.0001 and *P*>0.28 respectively). However, these similar patterns were associated with considerable differences between groups for each systolic phase and each tenting measure, so that tenting was consistently higher in LVD‐noMR versus controls and LVD‐FMR versus LVD‐noMR (Table 2). Furthermore, mean tenting height decline was greater in controls versus LVD‐noMR or LVD‐FMR (52% versus 25% and 21%, *P*<0.0001) but not different in both LVD groups (*P*=0.58). Similarly, tenting volume decline was greater in controls versus LVD‐noMR or LVD‐FMR (38% versus 21% and 14%, *P*<0.0001), but the difference between LVD groups did not reach significance (*P*=0.38). Thus, mitral valve position is dynamic in all groups of patients with similar patterns but with greater magnitude in normal subjects versus LVD. However, the main characteristic of LVD‐FMR is the largest tenting at all phases of systole.

**Table 2.** Effects of the Time Course in Systole on Dynamic Changes in Regurgitant Orifice Area

	Dynamic Changes in Functional Mitral Regurgitation			*P* Value
	Early‐Systole	Midsystole	Late‐Systole	
LVD‐FMR				
Phasic V_max_, cm/sec	310±59	481±66	314±44	<0.0001
Phasic regurgitant flow, mL/sec	120±53	106±44*	108±43	0.03
Phasic ERO, cm^2^	0.4±0.13	0.23±0.09	0.36±0.13	<0.0001
Maximum tenting length, mm	12.2±2.7§†	10.8±2.7§†║	10.7±2.8§†	<0.0001
Mean tenting length, mm	5.4±1.9§†	4.5±1.9§†║	4.2±1.6§†	<0.0001
Tenting volume, cm^3^	4.3±1.9§†	3.5±1.7§†║	3.6±1.7§†	<0.0001
LVD‐noMR				
Maximum tenting length, mm	10.2±2.8‡	8.1±2.8¶║	7.7±2.5¶	<0.0001
Mean tenting length, mm	3.8±1.5‡	2.8±1.5¶║	2.8±1.6¶	<0.0001
Tenting volume, cm^3^	2.9±1.5¶	2.2±1.2¶║	2.1±1.2¶	<0.0001
Controls				
Maximum tenting length, mm	6.9±2.0	4.4±2.1║	4.0±1.9	<0.0001
Mean tenting length, mm	2.6±1.2	1.2±1.1║	1.1±1.0	<0.0001
Tenting volume, cm^3^	1.4±0.8	0.8±0.6║	0.8±0.5	<0.0001

LVD‐FMR indicates left ventricle dysfunction and functional mitral regurgitation; LVD‐noMR, left ventricle dysfunction without functional mitral regurgitation; ERO, effective regurgitant orifice.

* *P*<0.05

^§^ *P*<0.001 low EF vs normal.

^†^ *P*<0.05,

^║^ *P*<0.001 functional MR vs low EF.

^‡^ *P*<0.05,

^¶^ *P*<0.001 functional MR vs normal.

In LVD‐FMR patients, the MR is also dynamic (Table 2). The peak velocity in midsystole is well established and the ERO has an opposite pattern with rapid decrease in midsystole from its early‐systolic peak and, in late‐systole subsequent increase towards its second peak. Conversely, because the driving force is higher in midsystole, the largest RVol occurred during that phase (20±9 mL) versus smaller volumes in early‐ and late‐systole (*P*<0.001). In analyzing simultaneous dynamic changes of MR and mitral valve, early‐ to midsystolic decline of ERO is associated with simultaneous decrease in tenting volume and increase in LV‐LA gradient (Table 2). Conversely mid‐ to late‐systolic increase in ERO occurs without change in tenting but with a decline in LV‐LA gradient only and may thus reflect declining closing pressure (Table 2).

### Mitral Annular Dynamics

**Figure 2.**
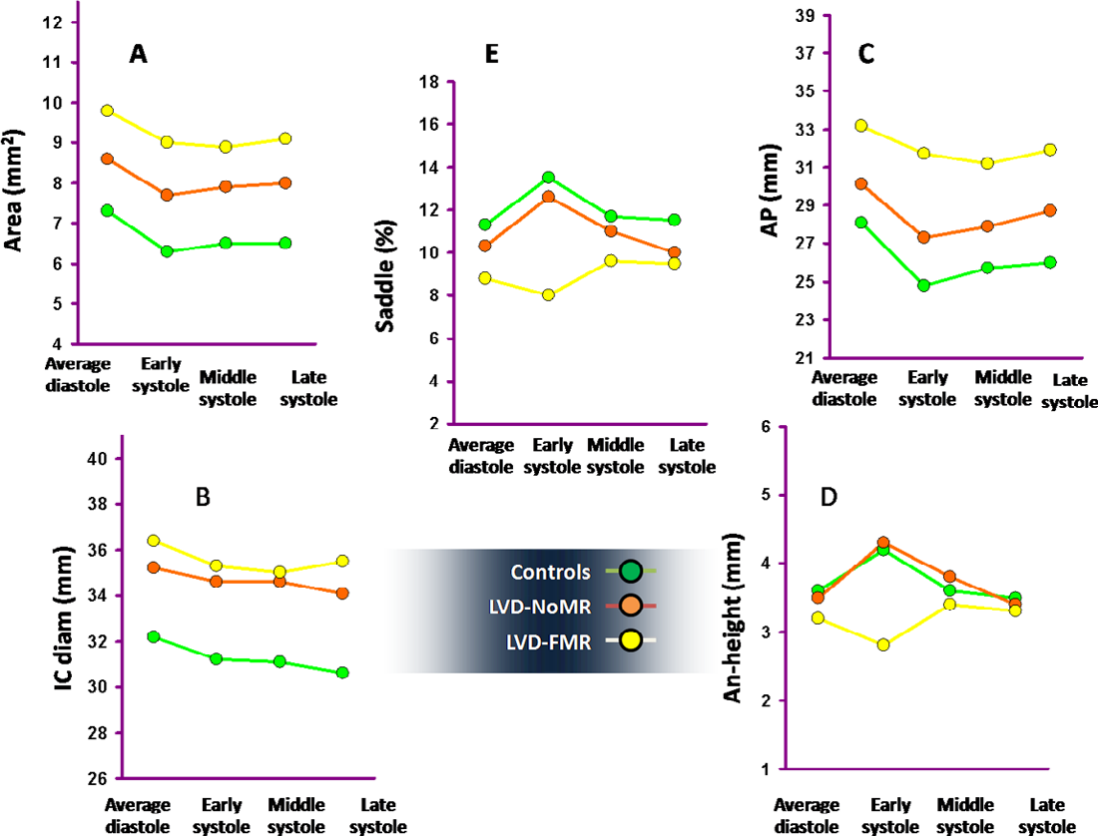
Mitral annular dimensions in each of the phases of the cardiac cycle are shown in normal control subjects, patients with low ejection fraction and no MR (LVD‐noMR) and patients with functional MR (LVD‐FMR). Because there were no systematic trends for change in annular dimensions during diastole in any group, average diastolic dimensions are shown. A, LVD‐FMR patients displayed larger annular area compared to controls and LVD‐noMR patients, throughout the cardiac cycle. B, LVD‐FMR and LVD‐noMR patients had similar inter commissural diameter, and both groups had larger intercommisural diameter compared to controls throughout the cardiac cycle. Intercommissural diameters did not change significantly during the entire cardiac cycle. C, Antero‐posterior diameter decreased and (D) annular height increased markedly in early‐systole with marked saddle‐shape (ratio of height to intercommissural diameter) accentuation (E) in both control and LVD‐noMR patients. On the other hand, in patients with LVD‐FMR antero‐posterior contraction was attenuated (C), annular height was unchanged (D), with a delayed and attenuated increase in midsystole, and annular saddle shape did not change and even tended to flatten in early‐systole. The within‐subject effects for all annular measurements apart for intercommisural diameter over time were significant (*P*<0.05). The interaction of group with time was significant only for annular height and saddle shape. LVD‐noMR indicates left ventricle dysfunction without functional mitral regurgitation; LVD‐FMR, left ventricle dysfunction and functional mitral regurgitation; AP, anterior papillary.

Mitral annular dimensions overall, as average of diastole and systole are shown in normal control subjects, LVD‐noMR and LVD‐FMR patients in Table 3. As expected, LVD‐FMR patients displayed larger annular circumference, area, antero‐posterior, and intercommissural diameters, and lower ratio of height to intercommissural diameter ratio (saddle‐shape depth) compared to controls. Compared with LVD‐noMR patients, LVD‐FMR patients displayed larger annular circumference, area, and antero‐posterior diameter, but similar intercommissural diameter and shallower saddle‐shape depth. Stratified by diastolic and systolic phases, annular area, circumference, antero‐posterior, and intercommissural diameters remained larger and saddle depth shallower in LVD‐FMR patients than in control subjects in both systole and diastole. However, this stratified comparison showed that the shallower annular height and saddle‐shape depth in patients with LVD‐FMR, was observed during systole alone. We compared each parameter to the same parameter in the following phase in the cardiac cycle and used stringent levels of significance (*P*<0.005 and *P*<0.001) to minimize the possibility of a false positive result. To avoid bias incurred by multiple comparisons we used the repeated measures linear model analysis to define the within‐group effect for each annular parameter over time, the between group differences over time, and the group by time interactions. The interactions of group with time were analyzed for the entire cohort and for each pair of groups (normal versus LVD‐noMR, normal versus LVD‐FMR and LVD‐noMR versus LVD‐FMR). Mitral annular dimensions averaged in each of the phases of the cardiac cycle are shown in normal control subjects, LVD‐noMR, and LVD‐FMR patients in Table 4 and Figure 2. Intercommissural diameters did not change significantly during the entire cardiac cycle in all groups, but antero‐posterior diameter decreased and annular height increased markedly in early‐systole with marked saddle‐shape (ratio of height to intercommissural diameter) accentuation in both the normal and LVD‐noMR groups but not in the LVD‐FMR group. These results are consistent with our previous report showing that the normal mitral annulus is dynamic.^15^ Although mitral annulus in LVD‐noMR subjects was larger in all dimensions compared to control patients, normal annular dynamics were maintained with no change in intercommissural diameters during the entire cardiac cycle, a significant decrease in anteroposterior diameter and increase in annular height in early‐systole with significant early‐systolic saddle‐shape accentuation. On the other hand, in LVD‐FMR patients, not only annular dimensions were larger compared to both of the other groups, but annular dynamics were abnormal. Diastolic to systolic antero‐posterior diameter changes were attenuated, annular height was unchanged, and annular saddle shape did not change in early‐systole. The interaction of group with time for annular height and saddle shape were both significant between the normal or LVD‐noMR groups and the LVD‐FMR group suggesting that annular dynamics with time are different between patients with functional MR and those without MR irrespective of systolic function.

**Table 3.** Annular Dimensions Overall, During Diastole and Systole Among Normal Subjects, Patients With Low EF and No MR and Patients With Functional MR

Measurement	All Cycle	Average Diastole	Average Systole
Annular area, cm^2^			
Normal	6.9±1.5	7.3±1.6	6.5±1.6
LVD‐noMR	8.2±2.0‡	8.5±2.1‡	7.8±1.9‡
LVD‐MR	9.4±2.0§†	9.8±2.2§†	9.0±2.0§†
AP diameter, mm			
Normal	26.9±3.1	28.1±3.2	25.5±3.2
LVD‐noMR	28.9±3.8	29.9±4.3	27.9±3.9‡
LVD‐MR	32.1±4.3§†	33.1±4.7§†	31.4±4.3§║
IC diameter, mm			
Normal	31.7±3.5	32.2±3.6	30.9±3.7
LVD‐noMR	34.8±4.2‡‡	35.2±4.3‡	34.5±4.5¶
LVD‐MR	35.8±3.6§	36.4±4.0§	35.2±3.6§
Circumference, mm			
Normal	94.4±10	97.5±10	90.9±11
LVD‐noMR	103.2±12‡	105.1±13‡	101.5±12¶
LVD‐MR	109.8±12§†	112.3±13§†	107.4±12§
Annular height, mm			
Normal	3.7±0.9	3.6±1.0	3.8±1.1
LVD‐noMR	3.7±1.0	3.5±1.4	3.9±1.1
LVD‐MR	3.2±1.3	3.1±1.5	3.1±1.5*†
Saddle shape%			
Normal	11.7±2.7	11.3±3.1	12.2±3.1
LVD‐noMR	10.8±3.0	10.1±3.9	11.5±3.2
LVD‐MR	8.8±3.5§†	8.7±4.3*	8.7±3.8§║

EF indicates ejection fraction; MR, mitral regurgitation; LVD‐FMR, left ventricle dysfunction and functional mitral regurgitation; LVD‐noMR, left ventricle dysfunction without functional mitral regurgitation; AP, anterior papillary.

* *P*<0.05,

^§^ *P*<0.001 low EF vs normal.

^†^ *P*<0.05,

^║^ *P*<0.001 functional MR vs low EF.

^‡^ *P*<0.05,

^¶^ *P*<0.001 functional MR vs normal.

**Table 4.** Annular Dimensions Throughout the Cardiac Cycle Among Normal Subjects, Patients With LVD‐NoMR and Patients With LVD‐MR

Measurement	Early‐Diastole	Middle‐Diastole	Late‐Diastole	Early‐Systole	Middle‐Systole	Late‐Systole	Within‐Subjects	Between‐Subjects	Time×Group Interaction
Annular area, cm^2^									
Normal	7.3±1.8	7.5±1.9	7.0±1.6.	6.3±1.6║	6.5±1.8	6.5±1.8	<0.0001	<0.0001	0.8
LVD‐noMR	8.7±2.1	8.6±2.3	8.3±2.1	7.7±2.0║	7.8±2.1	7.9±2.0			
LVD‐MR	9.7±2.2	9.8±2.3	9.8±2.3	9.0±2.1║	8.9±2.0	9.1±2.1			
AP diameter, mm									
Normal	28.4±3.7	28.5±3.6	27.3±3.5	24.8±3.5║	25.7±3.7	26.0±3.8	<0.0001	<0.0001	0.04*
LVD‐noMR	30.5±4.5	29.9±4.6	29.2±4.3	27.4±4.0║	27.8±4.0	28.4±4.2			
LVD‐MR	32.9±4.5	33.3±4.9	33.2±5.0	31.3±4.6	31.1±4.6	31.9±4.5			
IC diameter, mm									
Normal	31.7±4.3	32.3±4.9	32.1±3.7	31.2±4.2	31.1±4.4	30.6±4.2	0.06	<0.0001	0.8
LVD‐noMR	35.3±4.6	35.3±5.1	35.2±4.2	34.9±5.0	34.4±4.9	34.3±4.3			
LVD‐MR	36.3±4.8	36.2±4.2	36.5±4.4	35.0±4.1	35.2±4.0	35.4±3.9			
Circumference, mm									
Normal	97.4±12	98.8±13	95.6±10	91.3±11.2║	92.3±12.3	89.3±17.6	<0.0001	<0.0001	0.6
LVD‐noMR	106±13	106±15	103±13	101±13§	101±14	101±12			
LVD‐MR	111±13	112±13	112±13	107±12	107±13	108±12			
Annular height, mm									
Normal	3.8±1.3	3.7±1.4	3.3±1.0	4.2±1.2§	3.6±1.3	3.5±1.5	0.05	0.1	0.0005*†
LVD‐noMR	3.7±1.6	3.7±2.1	3.2±1.6	4.3±1.2§	4.0±1.6	3.5±1.4			
LVD‐MR	3.2±1.8	2.7±1.7*	3.6±2.2	2.8±1.9	3.1±1.8	3.3±1.5			
Saddle shape, %									
Normal	12.4±4.5	11.7±4.9	10.6±3.7	13.5±4.0║	11.7±4.5	11.5±4.5	0.03	0.0001	0.0009*†
LVD‐noMR	10.8±4.9	10.6±6.1	9.1±4.3	12.6±3.6║	11.7±4.6	10.3±4.2			
LVD‐MR	9.0±5.2*	7.6±4.7*†	10.0±6.4	8.0±5.2	8.8±5.3	9.2±4.4			

LVD‐noMR indicates left ventricle dysfunction without functional mitral regurgitation; LVD‐MR, left ventricle dysfunction and mitral regurgitation; AP, anterior papillary; IC, inter commissural.

* LVD‐FMR vs normal.

† LVD‐FMR vs LVD‐noFMR.

‡ LVD‐noFMR vs normal.

¶ *P*<0.005 within‐subject effect over time.

║ *P*<0.001 within‐subject effect over time.

An illustration of the difference in annular dynamics in controls and patients with functional MR is shown in Movies S1 and S2.

### Papillary Muscle Dynamics

The diameters of the triangle created between the antero‐lateral papillary muscle tip, postero‐medial papillary tip, and midanterior mitral annulus averaged over the entire cardiac cycle in control subjects, LVD‐noMR and LVD‐FMR patients are shown in Table 5.

**Table 5.** Interpapillary and Papillary to Annular Distance Overall, During Diastole and Systole Among Normal Subjects, Patients With Low EF and No MR and Patients With Functional MR

Measurement	All Cycle	Average Diastole	Average Systole
PM area, cm^2^			
Normal controls	2.9±0.9	3.1±0.9	2.8±1.1
LVD‐noFMR	4.8±1.1¶	4.8±1.2¶	4.9±1.1¶
LVD‐FMR	5.4±1.5§	5.4±1.6§	5.4±1.5§
PMP‐AA length, mm			
Normal controls	38.0±5.2	38.4±5.2	37.7±5.7
LVD‐noFMR	46.3±5.2¶	48.0±6.2¶	44.7±4.8¶
LVD‐FMR	50.0±5.2§†	50.4±6.0§	50.4±5.6§║
ALP‐AA length, mm			
Normal controls	35.8±5.6	36.1±5.4	35.5±6.1
LVD‐noFMR	42.5±5.5¶	43.4±5.8¶	42.0±5.1¶
LVD‐FMR	44.6±5.6§	44.3±6.3§	45.4±5.6§†
Inter PAP width, mm			
Normal controls	17.1±4.1	18.0±4.1	16.1±4.8
LVD‐noFMR	23.4±4.2¶	23.4±4.5¶	23.5±4.3¶
LVD‐FMR	25.1±4.9§	25.2±5.3§	25.2±5.0§
Inter PAP angle, °			
Normal controls	26.2±6.5	27.5±6.2	24.6±7.6
LVD‐noFMR	28.1±6.7	28.2±6.9	27.7±6.6
LVD‐FMR	28.9±6.3	29.0±7.0	28.7±6.0*
PPM/APM‐AA ratio			
Normal controls	1.07±0.09	1.08±0.09	1.07±0.1
LVD‐noFMR	1.08±0.07	1.10±0.09	1.07±0.06
LVD‐FMR	1.13±0.16	1.14±0.15*	1.12±0.15

LVD‐noMR indicates left ventricle dysfunction without functional mitral regurgitation; LVD‐FMR, left ventricle dysfunction and functional mitral regurgitation; EF, ejection fraction; PMP, postero‐medial papillary; ALP, antero‐lateral papillary; PPM, posterior papillary muscle; APM, anterior papillary muscle; AA, anterior annulus.

* *P*<0.05,

^§^ *P*<0.001 low EF vs normal.

^†^ *P*<0.05,

^║^ *P*<0.001 functional MR vs low EF.

^‡^ *P*<0.05,

^¶^ *P*<0.001 functional MR vs normal.

**Figure 3.**
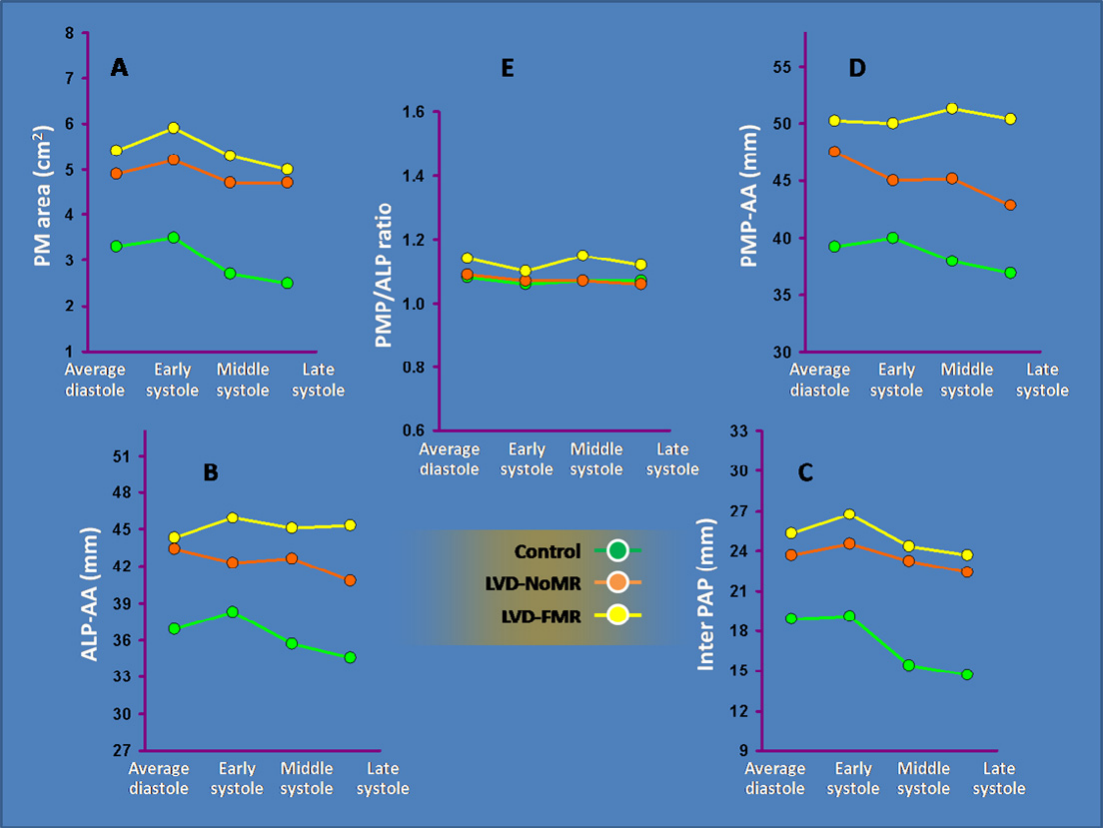
The area and diameters of the triangle created between the antero‐lateral papillary (ALP) muscle tip, postero‐medial papillary (PMP) tip and midanterior mitral annulus measured during the cardiac cycle in control subjects, LVD‐noMR and LVD‐FMR patients. A, Compared with LVD‐noMR patients, LVD‐FMR patients displayed similar ALP tip to anterior annulus (AA) distance (B) and interpapillary muscle distance (C) throughout the cardiac cycle. On the other hand, postero‐septal papillary tip to anterior annulus distance (D) was increased in LVD‐FMR patients, compared to both controls and LVD‐noMR patients throughout systole. In controls, papillary muscle tip motion in relation to the anterior annulus was characterized by symmetric antero‐lateral (B) inter papillary (C) and postero‐septal (D) papillary tip to anterior annulus approximation, preserving a constant ratio between those distances in midsystole (E). In LVD‐noMR patients, although postero‐septal and antero‐lateral papillary tip to anterior annulus approximation as well as midsystolic approximation between interpapillary tips were attenuated and delayed (B, C, D), the ratio between these distances was preserved throughout systole (E). On the other hand, in patients with LVD‐FMR, although midsystolic approximation between interpapillary tips (C), and ALP tip to AA motion (B) was similar to the motion in LVD‐noMR patients, postero‐medial tip paradoxically moved away from the anterior annulus in the first half of systole (D), resulting in an increase in the postero‐medial tip to anterior annulus and antero‐lateral tip to anterior annulus distance ratio in mid‐ and late‐systole (E). The within‐subject effect for the ratio of postero‐medial tip to anterior annulus and antero‐lateral tip to anterior annulus distance ratio was nonsignificant and constant throughout the cardiac cycle in the control and LVD‐noMR patients but not in the LVD‐FMR group. The between‐subject effect was significant for the postero‐medial tip to anterior annulus distance, and the postero‐medial tip to anterior annulus and antero‐lateral tip to anterior annulus distance ratio. The interaction of group with time was significant only for the postero‐medial tip to anterior annulus distance and the postero‐medial tip to anterior annulus and antero‐lateral tip to anterior annulus distance ratio. LVD‐noMR indicates left ventricle dysfunction without functional mitral regurgitation; LVD‐FMR, left ventricle dysfunction and functional mitral regurgitation; AP, anterior papillary.

Compared with control subjects, LVD‐FMR patients displayed larger triangular area, postero‐septal and antero‐lateral papillary tips to anterior annulus distance and interpapillary tips distance. Stratified by diastolic and systolic phases, all these parameters remained larger in LVD‐FMR patients than in control in both systole and diastole. Compared with LVD‐noMR patients, LVD‐FMR patients displayed similar triangular area, antero‐lateral papillary tip to anterior annulus distance, and interpapillary tips distance but larger postero‐septal papillary tip to anterior annulus distance. Stratified by diastolic and systolic phases, all the parameters were similar between these groups during diastole, but postero‐septal papillary tip to anterior annulus distance was larger in LVD‐FMR patients than in LVD‐noMR in systole alone. We compared each parameter to the same parameter in the following phase in the cardiac cycle and used stringent levels of significance (*P*<0.005 and *P*<0.001) to minimize the possibility of a false positive result. The results of the repeated measures linear model analysis to define the within‐group effect for each papillary muscle parameter over time, the between group differences over time, and the group by time interactions are presented in Table 6 and Figure 3. Normal papillary muscle tip motion in relation to the anterior annulus was characterized by shortening of interpapillary tips distance on the posterior left ventricular wall, decreased interpapillary angle, and symmetric postero‐septal and antero‐lateral papillary tip to anterior annulus approximation, preserving a constant ratio between those distances throughout the cycle.

**Table 6.** Interpapillary and Papillary to Annular Distance Throughout the Cardiac Cycle Among Normal Subjects, Patients With Low EF and No MR and Patients With Functional MR

Measurement	Early‐Diastole	Middle‐Diastole	Late‐Diastole	Early‐Systole	Middle‐Systole	Late‐Systole	Within‐Subjects	Between‐Subjects	Time×Group Interaction
PM area, cm^2^									
Normal controls	2.7±0.9	3.3±0.9*	3.4±1.2	3.5±1.2	2.7±1.6*	2.5±1.2	<0.0001	<0.0001	0.1
LVD‐noFMR	4.9±1.5	4.9±1.3	4.7±1.3	5.2±1.0	4.7±1.1*	4.9±1.5			
LVD‐FMR	5.5±1.8	5.2±1.9	5.5±1.7	5.9±1.9	5.3±1.5	5.1±1.6			
PMP‐AA length, mm									
Normal controls	37.6±6.6	39.8±5.6	38.5±6.1	40.0±6.5	37.9±6.9§	36.9±6.3	<0.0001	<0.0001	<0.0001*!
LVD‐noFMR	49.8±7.6*	47.7±6.2	46.8±6.7	45.6±7.2	45.5±5.9	42.9±5.9			
LVD‐FMR	50.8±6.6	50.4±6.5	50.4±7.1	50.0±6.0	51.3±6.8	50.4±6.5			
ALP‐AA length, mm									
Normal controls	35.0±6.2	37.9±6.1	35.8±6.0	38.3±7.9	35.6±7.7*	34.6±6.3	<0.0001	<0.0001	0.06
LVD‐noFMR	42.9±6.6	43.9±6.7	43.0±6.5	42.4±6.9	42.8±5.6	40.8±5.			
LVD‐FMR	44.4±6.6	44.2±7.7	45.1±6.3	46.0±6.6	45.1±6.5	45.3±5.9			
Inter PAP width, mm									
Normal controls	16.0±4.8	18.4±4.1	19.2±5.2	19.1±4.7	15.4±5.8*	14.7±5.7	<0.0001	<0.0001	0.05*!
LVD‐noFMR	24.2±5.3	23.0±4.7	22.9±5.5	24.5±4.3	23.2±4.8	22.9±5.6			
LVD‐FMR	25.4±6.1	25.0±6.6	25.3±6.3	26.9±6.3	24.5±5.5	23.9±5.6			
Inter PAP angle, °									
Normal controls	24.6±8.2	27.3±6.8	29.3±8.2	27.6±7.6	23.4±7.6*	23.2±9.7	<0.0001	0.2	0.3
LVD‐noFMR	27.5±7.6	28.1±7.1	28.3±8.2	30.6±8.3	27.0±6.7	26.0±7.0			
LVD‐FMR	29.0±8.0	28.4±9.0	29.2±7.6	31.0±7.6	27.8±6.5	26.8±7.8			
PPM/APM‐AA ratio									
Normal controls	1.08±0.15	1.07±0.13	1.07±0.10	1.06±0.14	1.07±0.13	1.07±0.10	0.01	0.05	0.01*
LVD‐noFMR	1.17±0.2	1.06±0.07	1.05±0.06	1.09±0.14	1.06±0.07	1.05±0.06			
LVD‐FMR	1.16±0.17	1.14±0.17	1.12±0.18	1.10±0.16	1.15±0.17§	1.12±0.18			

LVD‐noMR indicates left ventricle dysfunction without functional mitral regurgitation; LVD‐FMR, left ventricle dysfunction and functional mitral regurgitation; EF, ejection fraction; PMP, postero‐medial papillary; ALP, antero‐lateral papillary; PPM, posterior papillary muscle; APM, anterior papillary muscle; AA, anterior annulus.

* LVD‐FMR vs normal.

^†^ LVD‐FMR vs LVD‐noFMR.

^‡^ LVD‐noFMR vs normal.

^§^ *P*<0.005 within‐subject effect over time.

^║^ *P*<0.001 within‐subject effect over time.

In LVD‐noMR patients, there were no midsystolic approximation between interpapillary tips, postero‐septal and antero‐lateral papillary tip to anterior annulus approximation, preserving a constant ratio between these distances in midsystole. On the other hand, in patients with LVD‐FMR, although midsystolic approximation between interpapillary tips, postero‐medial tip and antero‐lateral papillary tip to anterior annulus motion was lost, just like in LVD‐noMR patients, there was an increase in the postero‐medial tip to anterior annulus and antero‐lateral tip to anterior annulus distance ratio in midsystole.

**Figure 4.**
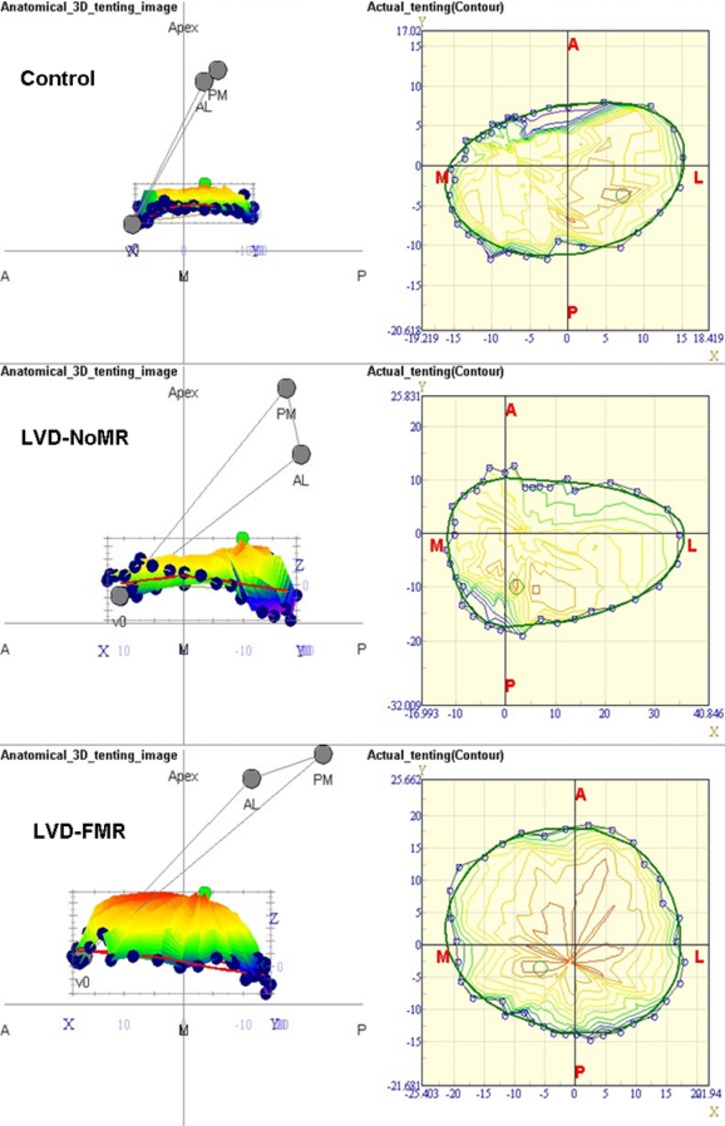
The dedicated software reconstructs anatomic 3D images of mitral apparatus in each phase including measurement of mitral annular area, annular circumference, maximal antero‐posterior diameter, maximal intercommissural diameter, leaflet maximal tenting volume, papillary muscle area (the area of the triangle formed between both papillary tips and midanterior annulus), length of posterior papillary tip to midanterior annulus, length anterior papillary tip to midanterior annulus, interpapillary tips distance, and the acute angle of the triangle formed between papillary tips and midanterior annulus. The figure shows the midsystolic images in an “oblique representation” (left images), and the “en face” view (right images). Tenting volume, annulus and papillary muscle position in controls (upper left figure), LVD‐noMR patients (middle left figure) and LVD‐FMR patients (lower left figure) are illustrated in the oblique representations, and mitral annular area, annular circumference, maximal antero‐posterior diameter, and maximal intercommissural diameter in the same groups are depicted in the “en face” (right) figures. In controls tenting volume is minimal, annulus has a deep saddle shape and papillary muscles are positioned symmetrically in front of the midanterior annulus (upper left figure). In midsystole the annulus assumes an elliptical appearance with maximal antero‐posterior diameter being smaller than intercommisural diameter due to the significant antero‐posterior contraction and annular folding in the intercommissural axis in early‐systole (upper right figure). In LVD‐noMR patients tenting volume is larger and so is the annulus and papillary muscles to midanterior annulus distances but the annulus maintains the normal deep saddle shape, the posterior and anterior papillary muscle to midannulus distances are similar and symmetric (middle‐left figure) and the annulus maintains an elliptical appearance (middle‐right figure). Importantly, the intersection of the maximal anterior to posterior dimension line and the medial to lateral dimension line is not at the center of the annulus, showing some distortion in annular shape which does not result in functional MR probably due to the maintained elliptical and saddle shape. In LVD‐FMR the annulus is flat and posterior papillary muscle to midanterior annulus distance is much longer than the distance between the anterior papillary muscle and midanterior annulus, both resulting in a much larger tenting volume than in LVD‐noMR patients (lower left figure). The annulus is circular with antero‐posterior diameter being similar to intercommissural diameter (lower right figure). LVD‐noMR indicates left ventricle dysfunction without functional mitral regurgitation; LVD‐FMR, left ventricle dysfunction and functional mitral regurgitation.

An example of midsystolic tenting volume, annulus, and papillary muscle position in controls, LVD‐noMR patients, and LVD‐FMR patients is illustrated by the report produced by the dedicated quantification software is shown in Figure 4.

### Multivariable Analysis

We used stepwise multivariable analyses to explore the contribution of saddle‐shape accentuation and papillary muscle position on MR in the entire population. As shown in Table 7, less saddle shape (or in other words, flatter annulus) in early‐systole contributed significantly to worse early‐systolic ERO even after adjustment for annular area, systolic blood pressure, and tenting volume, but did not have any effect on midsystolic ERO. Symmetric papillary muscle position had significant impact on midsystolic ERO after adjustment to annulus area, systolic blood pressure tenting volume and saddle shape, but did not have any effect on early‐systolic ERO. We have assessed the role of ventricular closing forces (early‐ and midsystolic transmitral gradient) on severity of functional MR by adding those parameters to the models. Early‐ and midsystolic transmitral gradients did not have any added role on severity of functional MR in the entire cohort (*P*>0.5 for both). Only 2 of the LVD‐FMR patients had EF>50% and extensive regional wall motion abnormalities, while all the patients in the LVD‐noMR group had EF<50%. Although the difference in EF might have resulted in different influence of closing forces in those 2 patients, we lacked any statistical power to demonstrate it due to the minimal amount of such patients.

**Table 7.** Multivariable Analyses to Explore the Contribution of the Different Parameters on Early‐Systolic and Midsystolic ERO

	Sample Size N=120 Patients	
	Early‐Systolic ERO Multivariate Regression Coefficient±SE; *P*‐Value	Midsystolic ERO Multivariate Regression Coefficient±SE; *P*‐Value
Annulus area	−0.002±0.01; *P*=0.9	0.004±0.005; *P*=0.47
Tenting volume	0.06±0.01; *P*<0.0001	0.03±0.005; *P*<0.0001
Saddle shape	−0.01±0.003; *P*=0.001	−0.002±0.002; *P*=0.11
PPM/APM to AA distance ratio	0.16±0.18; *P*=0.15	0.3±0.07; *P*=0.0001
Systolic blood pressure	−0.001±0.0007; *P*=0.09	−0.0004±0.0005; *P*=0.11
*P* value	<0.0001	<0.0001
R^2^*	0.43	0.42

ERO indicates effective regurgitant orifice; PPM, posterior papillary muscle; APM, anterior papillary muscle; AA, anterior annulus.

* Model fit validation was performed by graphical residual analysis. The overall model fit had R^2^=0.43 for the early‐ERO and R^2^=0.42 for the midERO models. The residuals demonstrated normal distribution for both models.

### Subgroup Analysis

#### Etiologies of systolic dysfunction

The LVD‐FMR and LVD‐noMR groups were heterogeneous in terms of etiology of systolic dysfunction with the LVD‐FMR group having a greater proportion of patients with ischemic cardiomyopathy than the LV‐noMR group, which could have caused the difference observed in the symmetry and coordination of papillary muscle to the annular distance. We grouped the patients on the basis of etiology of systolic dysfunction: global LV dysfunction (38 patients, 16 LVD‐FMR and 22 LVD‐noMR), anterior MI (25 patients, 17 LVD‐FMR and 8 LVD‐noMR) and inferior MI (15 patients, 10 LVD‐FMR and 5 LVD‐noMR) and re‐examined the averaged systolic data and early‐ and midsystolic data within each group according to the presence of mitral regurgitation.

Analyzing averaged systolic data in the patients with anterior MI**,** LVD‐FMR patients displayed larger annular area (*P*=0.04), antero‐posterior diameter (*P*=0.03), a trend for larger circumference (*P*=0.07), and lower saddle‐shape depth (9.5±4.2 versus 11.5±2.9; *P*=0.1), but no significant differences in intercommissural diameters or annular height compared to patients with anterior MI and no MR. Patients with anterior MI and FMR had significantly flatter early‐systolic saddle shape (7.9±4.8 versus 13.8±3.5; *P*=0.008), but the ratio of postero‐septal and antero‐lateral papillary tip to anterior annulus remained constant throughout systole, compared to the LVD‐noMR patients with anterior MI. In patients with global dysfunction, LVD‐FMR patients displayed a strong trend for larger annular area (8.5±2.3 versus 7.8±2.1), antero‐posterior diameter (30.2±4.9 versus 27.4±4.0), and lower saddle‐shape depth (9.6±4.1 versus 11.3±3.1; *P*=0.1), but no differences in intercommissural diameters, circumference, or annular height compared to patients with global dysfunction and no MR. Patients with global dysfunction and FMR had significantly flatter early‐systolic saddle shape (9.1±5.5 versus 12.2±3.9; *P*=0.04), but the ratio of postero‐septal and antero‐lateral papillary tip to anterior annulus remained constant throughout systole, compared to patients with global dysfunction and nonMR. In patients with inferior MI, LVD‐FMR patients had similar annular area, antero‐posterior and intercommissural diameters, but a strong trend for lower saddle‐shape depth (7.1±3.4 versus 11.3±3.9; *P*=0.08), and larger distances between the posterior papillary muscle and the anterior annulus, compared to patients with inferior MI and no MR. Patients with inferior MI and FMR had flatter early‐systolic saddle shape (7.0±5.6 versus 12.8±3.4; *P*=0.03), and the ratio of midsystolic postero‐septal and antero‐lateral papillary tip to anterior annulus was higher than in patients with inferior MI and no FMR (1.3**±**0.2 versus 1.0±0.02; *P*=0.02).

Multivariable analyses to explore the contribution of saddle‐shape accentuation, papillary muscle asymmetry, annular area, and tenting volume on early‐ and midsystolic MR subgrouped to patients with anterior MI, global dysfunction and inferior MI have shown that those determinants differed between the different etiologies. The determinants of early‐systolic MR in patients with anterior MI were larger annular area (0.07±0.03, *P*=0.03) and flatter early‐systolic saddle shape (‐0.02±0.0008, *P*=0.02), but not papillary muscle asymmetry. In patients with global dysfunction the only significant determinant of early‐MR was flatter saddle shape (−0.02±0.008, *P*=0.05). In patients with inferior MI the only determinant of early‐systolic MR was tenting volume (0.13±0.03, *P*=0.004), but none of the annular parameters.

The only determinant of midsystolic MR in patients with anterior MI was annular area (0.04±0.01; *P*=0.02). The determinants of midsystolic MR in patients with global dysfunction were larger tenting volume (0.03±0.01, *P*=0.02) and asymmetric papillary muscle position (0.44±0.18, *P*=0.02). In patients with inferior MI the only determinant of midsystolic MR was asymmetric papillary muscle position (0.34±0.1, *P*=0.01).

#### Intraventricular dys‐synchrony

Forty‐five patients had intraventricular dys‐synchrony (3 normal, 26 in the LVD‐FMR group and 16 in the LVD‐noMR group). They had attenuated saddle‐shape deepening at early‐systole (9.2±4.8 versus 12.5±4.7; *P*=0.006), but not at mid‐ or late‐systole (*P*>0.2 for both). Although patients with intraventricular dys‐synchrony had significantly longer anterior and posterior papillary muscle to annulus distances throughout systole (*P*<0.01 for all measurements), the ratio of posterior and anterior to annulus distances in early‐, mid‐ and late‐systole was similar compared to patients with normal conduction.

We have reanalyzed the models in the subgroups of patients with and without intraventricular dys‐synchrony. The determinants of early‐ and midsystolic MR remained tenting volume (0.05±0.02, *P*=0.05) and flatter annular saddle shape (−0.02±0.007, *P*=0.05) in early‐systole, and tenting volume (0.04±0.01, *P*=0.03) and increased posterior papillary to anterior papillary muscle distance ratio (0.43±0.13, *P*=0.002) in midsystole in patients with dys‐synchrony.

### Mitral Annular Dimensions Intra‐ and Interobserver Variability

Comparison of intraobserver parameters showed good agreement between measurements: annular area (mean difference 0.07±0.23 cm^2^ r=0.96; *P*=0.77), tenting volume (mean difference 0.11±0.12 cm; r=0.97; *P*=0.38), and annular height (mean difference 0.3±0.4 mm; r=0.54; *P*=0.47). The Bland‐Altman plot showed a random scatter of points around 0, indicating no systematic bias or measurement error proportional to the measurement value. Measurement variability (within‐subject coefficient of variation and 95% confidence interval of the Bland‐Altman method) for measurements for intraobserver differences was as follows: annular area 2.7% and ±0.23 cm; tenting volume, 5.4% and ±0.12 cm; and annular height, 14.3% and ±0.4 mm. Comparison of interobserver parameters showed good agreement between measurements as well: annular area (mean difference 0.04±0.23 cm^2^; r=0.97; *P*=0.87), tenting volume (mean difference 0.2±0.4 cm; r=0.79; *P*=0.67); and annular height (mean difference −0.26±0.38 mm; r=0.68; *P*=0.50). The Bland‐Altman plot showed a random scatter of points around 0, indicating no systematic bias or measurement error proportional to the measurement value. Measurement variability (within‐subject coefficient of variation and 95% confidence interval of the Bland‐Altman method) for measurements for interobserver differences was as follows: annular area 2.8% and ±0.23 cm; tenting volume, 12.9% and ±0.4 cm; and annular height, 11.8% and ±0.38 mm.

## Discussion

The present study aimed to define mitral annular and papillary muscle to annulus dynamics using RT3DE technology, and to define whether they help preserve mitral competence.

Our results show that (A) Functional MR is associated with LV remodeling and enlargement, as described by others.^1,7–8^ (B) Normal annulus is dynamic, with prominent annular folding across the intercommissural axis in early‐systole, and the loss of normal annular contraction contributes to early‐mitral incompetency in patients with systolic dysfunction. (C) Papillary muscle dynamic changes are linked to the valve deformation dynamics and to the severity of functional MR in mid‐ to late‐systole. (D) The dynamic changes differ in patients with different etiologies for functional MR. (E) Functional MR is created by a complex dynamic change of the valvular‐ventricular interaction, an observation that may have important implications for evolving per‐cutaneous and surgical treatments of FMR.

### 3D Dynamics of the Normal Mitral Annulus

Our findings using new RT3DE to assess normal mitral annulus confirm the results of previous studies using quantification attempts in normal subjects with 2D^8,23^ and 3D reconstruction,^15,24–26^ demonstrating the sequence of normal mitral annulus conformational changes over the cardiac cycle. Variation of annular size throughout diastole was minimal, as previously described.^17^ As we have previously shown,^15^ normal annulus saddle shape is accentuated in early‐systole, coinciding with iso‐volumic contraction. Simultaneously, annular antero‐posterior contraction and increased height occur without intercommissural change. Early‐systolic antero‐posterior folding across the fixed intercommissural diameter leads to early‐systolic annular area contraction and approximates anterior and posterior leaflets. Thus, in the normal valve, when early‐systolic ventricular pressure is relatively low and does not yet press the leaflets together,^8^ leaflet approximation by annular contraction may contribute to their coaptation^15^ and be temporarily important in preventing regurgitation (Movie S1). This early–annular antero‐posterior contraction, and saddle‐shape accentuation, has been postulated to be owing to either expansion of the aortic root in systole, thought to bring about folding by displacing the aorto‐mitral curtain posteriorly away from the aorta,^24,27^ or tethering of the anterior annulus to the aortic root combined with apical translation of the entire annulus resulting in folding across the intercommissural axis.^28–29^ Our analysis shows that this very early–annular folding begins during the iso‐volumic contraction, preceding aortic ejection, thus suggesting a major role for the second mechanism which does not depend on ejection and aortic displacement. Once leaflets are firmly opposed by early–annular folding, late‐systolic changes in annular area have little potential for inducing MR because of the firm apposition of the leaflets by increasing intraventricular pressure. Thus, improved 3D technology provides new insights into normal annulus dynamics, the complex changes of which converge toward mitral valve competence throughout systole.

### 3D Dynamics of Mitral Annulus in Patients With Low Ejection Fraction With and Without FMR

Compared with control subjects, the annulus in LVD‐noMR patients, as well as in patients with LVD‐FMR was larger throughout the cardiac cycle. However, comparison of annular dimensions in patients with low EF, with and without FMR, shows that not all annular enlargements are identical. Indeed, although intercommissural diameter was similar in LVD‐FMR and LVD‐noMR patients, LVD‐FMR patients displayed enlarged antero‐posterior diameter compared to LVD‐noMR patients. Although all groups had similar diastolic saddle shape, early‐systolic annular folding, and saddle‐shape deepening was absent in LVD‐FMR patients but not in LVD‐noMR patients.

In LVD‐FMR, annular height and intercommissural diameter changes are minimal leading to an adynamic annulus in term of saddle shape (Movie S1) as opposed to LVD‐noMR patients, in which normal early‐systolic annular folding and saddle‐shape deepening is achieved as a result of concomitantly increased annular height and unchanged intercommissural diameter. Annular shape differences are associated with contraction differences as well. The annulus in LVD‐FMR patients does not contract in early‐systole both in the antero‐posterior and intercommissural axes. Conversely, in both control and LVD‐noMR patients, annulus contracts in early‐systole only in the antero‐posterior dimension, leading to normal early‐systolic approximation of anterior and posterior mitral leaflets.

In conclusion, the loss of annular folding across the intercommissural axis and deepening of annulus in early‐systole, may play a role in early‐systolic FMR^17,24,30–31^ just as it is in myxomatous mitral valve disease^15^ and cardiomyopathy.^26,30^

### Papillary Muscle to Annulus Motion in Normal Subjects

Our findings using RT3DE throughout the cardiac cycle also offer new insights into normal papillary motion in relation to the mitral annulus and to its contribution to firm mitral valve sealing. During the first half of systole papillary muscles move closer together and move concurrently towards the mitral annulus with a well coordinated and symmetric motion, maintaining equal distances between the postero‐medial papillary muscle, antero‐lateral papillary muscle andmid anterior mitral annulus, avoiding distortion of mitral leaflets.

The systolic papillary to annulus approximation in the first half of systole follows the very early‐systolic annular contraction, and folding which result in leaflet approximation. Thus, in the normal valve, when early‐systolic ventricular pressure is relatively low leaflet approximation by annular contraction and folding is a major contributor to coaptation,^15^ but once leaflets are firmly opposed by the early–saddle‐shape accentuation, firm apposition of the leaflets by increasing intraventricular pressure is aided by the coordinated and symmetric descent of papillary muscles towards the annulus.

### 3D Dynamics of Papillary to Annulus Movement in Patients With Low Ejection Fraction With and Without FMR

In patients with low ejection fraction, irrespective of FMR, interpapillary muscle approximation is attenuated. This is possibly due to reduced circumferential and radial basal contraction in patients with systolic dysfunction. On the other hand, there are marked differences between patients with and without FMR, in terms of the postero‐medial papillary muscle, and antero‐lateral papillary muscle movement towards the midanterior mitral annulus.

In LVD‐noMR patients postero‐medial papillary muscle, and antero‐lateral papillary muscle to midanterior mitral annulus approximation is suppressed, but the distances between the postero‐medial papillary muscle, antero‐lateral papillary muscle and midmitral annulus remain equal, avoiding excessive midsystolic tethering and distortion of mitral leaflets. In contrast to the suppressed but symmetric motion of papillary muscles towards the midanterior annulus in LVD‐noMR patients, in patients with FMR postero‐medial muscle tip tends to paradoxically move away from the annulus.

Thus, as opposed to the normal valve, irrespective of ejection fraction, in which firm apposition of the leaflets by increasing intra‐ventricular pressure is aided by the interpapillary approximation and symmetric descent of papillary muscles towards the annulus, in patients with FMR, asymmetric midsystolic postero‐medial papillary muscle displacement results in abnormal tethering geometry.

### Changing Mechanisms for Functional Mitral Regurgitation in the Cardiac Cycle

Our large series of patients with quantified FMR, stratified by the time during the cardiac cycle, shows that valvular and sub‐valvar determinants of lesion severity (ERO) of FMR are not uniform throughout the cardiac cycle. Very early in systole, a flatter annulus, which does not assume the normal saddle shape contributes significantly to worse early‐systolic ERO, even after adjustment for known contributors for FMR as dilated annular area and increased tenting volume. We believe that preserved very early‐systolic antero‐posterior contraction (annular folding), with fixed intercommissural diameter in patients with low ejection fraction may approximate anterior and posterior leaflets. Thus, in the dysfunctional left ventricle, when early‐systolic ventricular pressure is relatively low and does not yet press the leaflets together,^8^ leaflet approximation by annular contraction and folding may contribute to their coaptation^15^ and be temporarily important in preventing early‐regurgitation.

Conversely, later in systole a flat annulus, while present, is of less consequence. Thus, another factor causing FMR is required to perpetuate the regurgitant lesion (ERO) in mid‐ and late‐systole. In the second and third parts of systole, asymmetric papillary muscle motion towards the midanterior annulus results in leaflet deformation with increased tenting volume, preventing appropriate coaptation,^32^and is the main determinant of ERO. Thus, our quantitative data shows that early‐systolic FMR and mid‐ to late‐systolic FMR result from 2 distinct mechanisms; a non dynamic and flat annulus in early‐systole and non ‐coordinated papillary to anterior annulus motion resulting in leaflet deformation with tenting in mid‐ and late‐systole.

### Subgroup Analysis

Analysis of the mechanism of early‐ and late‐ to midsystolic MR in patients with anterior MI, global dysfunction and inferior MI shows that those mechanisms differ depending on the etiology of LV systolic dysfunction. Early in systole, a flatter and larger annulus, which does not assume the normal “deep” saddle shape contributes significantly to worse early‐systolic MR, in patients with anterior MI and global dysfunction but is less important in patients with inferior MI in which MR depends entirely on tenting volume. On the other hand, in midsystole MR depends only on annular area in patients with anterior MI, but on symmetry and coordination of papillary muscle motion in both inferior MI and global dysfunction patients.

### Limitations

Other mitral measurements can be considered for valve anatomy and regurgitation. Unfortunately, in the present study, we use an older version of the RealView software, which does not estimate tenting area, thus minimal required surface for closure, available leaflet area, and coaptation area which can have profound effects on coaptation leading to MR cannot be analyzed. The FMR and LVD‐noMR groups are small, thus sub group analyses for different etiologies for LV dysfunction (inferior versus anterior ischemia versus global dysfunction) or dys‐synchrony versus normal conduction are limited, and we may lack the statistical power to assess the impact on MR of some potentially important parameters in those subgroups. However, our study is the largest RT3D trans‐thoracic echocardiography study to date, and suggests that future prospective studies with technically challenging sequential valvular, annular, and MR measurements should shed light on the mechanism by which FMR repair succeeds or fails.

### Clinical Perspective

Functional mitral regurgitation is frequent, portends poor prognosis underscoring the importance of comprehending its determinants. Although it is unquestionably associated with LV remodeling and enlargement, it is also affected by a complex dynamic change of annular and valvular‐ventricular interactions which have been poorly defined. To address these issues, we prospectively quantified MR in patients with functional MR and compared them with patients with normal heart or LV dysfunction and no MR. We defined mitral annular and papillary muscle to annulus dynamics using 3D technology 6 times during the cardiac cycle, and assessed whether these dynamics help preserve mitral competence. We show that the loss of normal annular folding across the intercommissural line and the resulting loss of saddle shape “deepening” contributes to early‐mitral incompetency, especially in patients with global systolic dysfunction, and/or anterior MI. We also show that asymmetric papillary muscle position is linked to the valve deformation and to the severity of functional MR in mid‐ to late‐systole, more so in patients with inferior MI. These new insights may lead to refined and individualized concepts for functional mitral regurgitation repair techniques. For instance, in patients with asymmetric papillary muscle position adjunctive procedures that improve valvular‐ventricular relations like strut chord transaction, papillary muscle repositioning, or infarct placation may be used. On the other hand, in patients with deranged annular motion newly introduced surgical approaches using flexible rings, preserving mitral annular function may be preferred. Therefore, for optimal surgical management of patients with functional MR, careful evaluation of annular and papillary motion throughout systole using advanced 3D technology may lead to refined concepts for functional mitral regurgitation patho‐physiology and repair techniques.

## Conclusions

FMR is affected by a complex dynamic change of the valvular‐ventricular interaction. It is linked to loss of annular contraction across the intercommissural axis (annular folding) that results in loss of early‐systolic mitral competence, and to papillary muscle dynamic changes that are linked to the valve deformation dynamics and to the severity of MR in mid‐ to late‐systole. These new insights should lead to refined concepts for functional mitral regurgitation pathophysiology and repair techniques.
